# Supplementary figures and images for: Boredom Makes Me Sick: Adolescents’ Boredom Trajectories and Their Health-Related Quality of Life
Source: Int J Environ Res Public Health. 2021 Jun 10;18(12):6308. doi: 10.3390/ijerph18126308 (PMC8296113; doi:10.3390/ijerph18126308)

Boredom relationship with HRQoL across genders

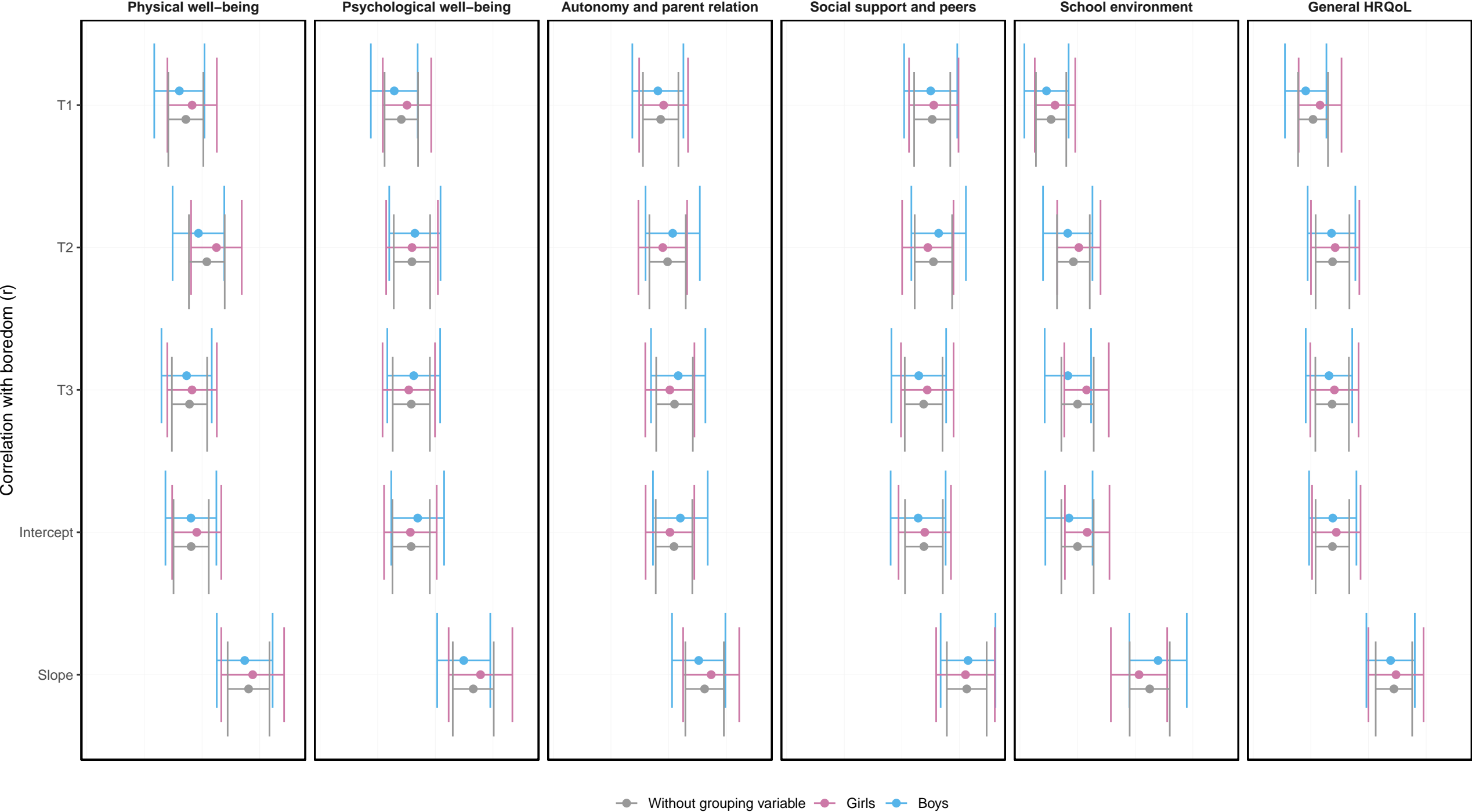

Supplement: Supplementary file 1 [file ijerph-18-06308-s001.zip › ijerph-1209093-supplementary.pdf]
